# Appropriate utilization of cardiac computed tomography for the assessment of stable coronary artery disease

**DOI:** 10.1186/s12872-021-01957-z

**Published:** 2021-03-26

**Authors:** Michael Hammer, Muhtashim Mian, Levi Elhadad, Mary Li, Idan Roifman

**Affiliations:** 1 Sunnybrook Health Sciences Centre, University of Toronto, Toronto, Canada; 2 Institute of Health Policy Management and Evaluation, University of Toronto, Toronto, Canada; 3 Medicine and Medical Imaging, Adjunct Scientist, Institute for Clinical Evaluative Sciences, Sunnybrook Health Sciences Centre, University of Toronto, 2075 Bayview Avenue, Room M315, Toronto, ON M4N-3M5 Canada

**Keywords:** Cardiac computed tomography, Coronary artery disease, Appropriate use criteria, Value of non-invasive testing

## Abstract

**Background:**

Appropriate use criteria (AUC) have been developed in response to growth in cardiac imaging utilization and concern regarding associated costs. Cardiac computed tomography angiography (CCTA) has emerged as an important modality in the evaluation of coronary artery disease, however its appropriate utilization in actual practice is uncertain. Our objective was to determine the appropriate utilization of CCTA in a large quaternary care institution and to compare appropriate utilization pre and post publication of the 2013 AUC guidelines. We hypothesized that the proportion of appropriate CCTA utilization will be similar to those of other comparable cardiac imaging modalities and that there would be a significant increase in appropriate use post AUC publication.

**Methods:**

We employed a retrospective cohort study design of 2577 consecutive patients undergoing CCTA between January 1, 2012 and December 30, 2016. An appropriateness category was assigned for each CCTA. Appropriateness classifications were compared pre- and post- AUC publication via the chi-square test.

**Results:**

Overall, 83.5% of CCTAs were deemed to be appropriate based on the AUC. Before the AUC publication, 75.0% of CCTAs were classified as appropriate whereas after the AUC publication, 88.0% were classified as appropriate (*p* < 0.001). The increase in appropriate utilization, when extrapolated to the Medicare population of the United States, was associated with potential cost savings of approximately $57 million per year.

**Conclusions:**

We report a high rate of appropriate use of CCTA and a significant increase in the proportion of CCTAs classified as appropriate after the AUC publication.

## Background

The Appropriate Use Criteria (AUC) were introduced by the American College of Cardiology Foundation in response to growing concern regarding potential overutilization of non-invasive cardiac imaging [1–3]. The aim of the AUC is to provide guidance to physicians with diagnostic test selection while optimizing scarce resources and healthcare expenditures [4]. As a result of the Protecting Access to Medicare Act, starting January 1st, 2021, physicians in the United States will be required to consult the AUC prior to ordering advanced imaging studies such as magnetic resonance imaging and computed tomography studies [5]. Similar other jurisdictions, including several Canadian provinces, are also considering implementing an appropriateness-based approach to medical imaging utilization.

Cardiac computed tomography has become an increasingly utilized imaging modality for a variety of indications, with assessment of coronary artery disease by angiography (cardiac computed tomography angiography; CCTA) being the most common [6, 7]. Prior research reported that up to 48% of CCTA referrals may be inappropriate [8, 9]. It is currently unclear if publication of the AUC has led to increased rates of appropriate CCTA utilization. There is a paucity of data comparing the rates of appropriate utilization of CCTA before and after publication of the AUC [9–13].

In this study, we sought to determine the proportion of appropriate CCTA utilization in a quaternary care institution pre- and post-publication of the AUC and to identify a potential shift toward more appropriate use of CCTA. In addition, we aimed to estimate potential cost savings associated with this potential shift. We hypothesized that there would be a significantly higher percentage of CCTAs deemed to be appropriate when ordered after, compared to before, the publication of the AUC and that there will be a significant cost savings associated with increased appropriate use.

## Methods

### Study design

**Fig. 1:**
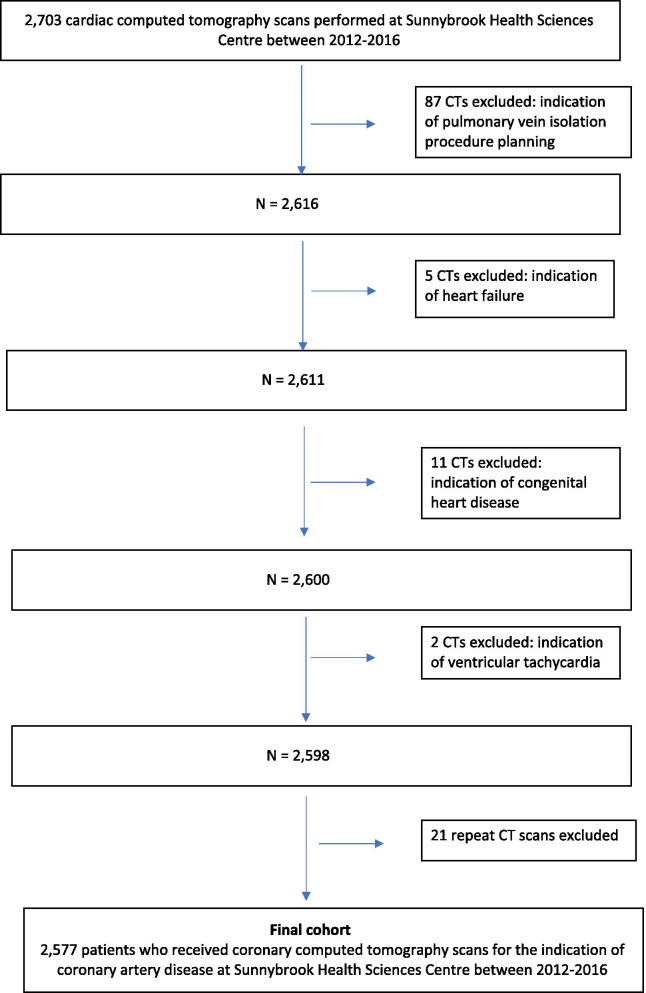
Derivation of the patient population

We conducted a retrospective cohort analysis of consecutive patients undergoing CCTA for the evaluation of coronary artery disease between January 1, 2012 and December 30, 2016 at Sunnybrook Health Sciences Centre, a large quaternary care Canadian medical centre. These patients were referred from 168 referring physicians. Of these referring physicians, 138 (82%) were cardiologists, 21 (13%) were internal medicine specialists and 9 (5%) were cardiovascular surgeons. Because we aimed to include only those patients who underwent coronary computed tomography angiography for the indication of CAD (i.e. CCTAs), patients receiving cardiac CTs for other indications were excluded (see Fig. 1). If a patient received more than one CCTA during our study period, only their first CCTA was included in our cohort. Further, pre-procedural CT scans performed for the pre-operative evaluation for trans-catheter aortic valve replacement (TAVR) and minimally invasive coronary artery bypass grafting were also excluded. These CT scans are not considered cardiac CTs at our institution and are instead classified as either ‘TAVR aortic CT’ scans for pre-operative TAVR evaluation and ‘MICAB’ for pre-operative evaluation for minimally invasive coronary artery bypass grafting. The study was approved by the local Research Ethics Board. While this study was funded by the Heart and Stroke Foundation of Canada (HSFC), HSFC had no role in the design of the study and in the collection, analysis, and interpretation of the data and in the writing of the manuscript.

### Identification of participants and data collection

Indications and procedure dates of patients undergoing CCTA were obtained from billing records compiled at the local Department of Medical Imaging. Each medical chart was subsequently reviewed by one of the authors (LE) to confirm that the test was indeed performed as well as its timing. Demographic information and indication for the CCTAs were obtained from patient charts, prior imaging reports and information on the medical imaging test requisition. Following data collection, appropriateness of each CCTA was classified categorically as “appropriate”, “may be appropriate” or “rarely appropriate” in a manner previously described and validated [11, 14–16]. In instances where information required to determine appropriateness was lacking, additional details were obtaining by contacting the ordering physician. The 2013 Multimodality Appropriate Use Criteria for the Detection and Risk Assessment of Stable Ischemic Heart disease was used as the AUC publication of reference [16].

### Statistical analysis

Descriptive statistics were utilized to describe the patient population. Characteristics of the patient population were compared using the chi square test for categorical values and the Mann–Whitney test for continuous variables. Appropriateness categories were compared between pre- and post- AUC publication groups via the chi-square test for both the overall cohort as well as for the subgroups of symptomatic and asymptomatic patients. A two tailed *p* value of < 0.05 was used as an indication of statistical significance. All statistical analyses were performed using SAS version 9.3 (Cary, NC).

### Cost analysis

We could not identify reliable estimates for the number of CCTAs performed for the entire population of the United States. Further, there exist re-imbursement discrepancies amongst different private insurance plans. Thus, we elected to estimate cost savings for the Medicare population of the United States (those aged 65 years and older). Estimates of unit costs for CCTA and absolute number of CCTAs performed were determined via the 2018 Medicare re-imbursement rate and based on prior research reporting the temporal trends in utilization for CCTA amongst the Medicare population [17–19]. Potential cost savings derived from the AUC publication were estimated as follows. Pre and Post AUC costs for rarely appropriate studies were estimated by multiplying the percentage of rarely appropriate CCTA scans by the estimated number of CCTAs performed in the Medicare population of the United States and by the unit costs per CCTA scan. Cost savings were then calculated as the difference between the post and pre AUC costs for these rarely appropriate studies.

## Results

### Patient population

A total of 2,577 consecutive CCTA patient files from January 1, 2012 and December 30, 2016 were reviewed. 889 of these CCTAs were performed prior to the 2013 AUC publication while 1688 were performed after the publication.

### Patient demographics

The patient demographics are presented in Table 1. The mean age was 59.7 years and approximately 47% of the patients were female. Regarding cardiac risk factors, 20.1% of patients had a family history of coronary artery disease, 14.1% were active smokers, 43.4% had diabetes, 37.6% had dyslipidemia and 18.8% had hypertension. 16.7% had prior history of cerebrovascular disease or peripheral vascular disease and 9% had newly diagnosed systolic heart failure. Typical anginal chest pain was present in 20.6% of patients, atypical chest pain in 66.6% of patients and there was no chest pain reported in 12.7% of patients. 22.3% of patients had an abnormal stress test prior to their CCTA. There was no significant difference in any of these patient characteristics pre and post AUC publication (*p* ≥ 0.05, see Table 2).

**Table 1:** Characteristics of the patient population who underwent cardiac CT for the evaluation of coronary artery disease; 2012–2016

n	2577
Age (mean ± SD in years)	59.7 ± 12.5
Female sex (%)	47.3
Cardiac risk factors	
Family history of CAD (%)	20.1
Active smoker (%)	14.1
Diabetes (%)	43.4
Dyslipidemia (%)	37.6
Hypertension (%)	18.8
Newly diagnosed systolic heart failure (%)	9.0
Prior history of cerebrovascular or peripheral vascular disease (%)	16.7
Chest pain	
Typical (%)	20.6
Atypical (%)	66.6
No chest pain (%)	12.7
Atrial fibrillation (%)	10.9
History of sustained ventricular tachycardia (%)	1.1
Abnormal prior stress test (%)	22.3

*CT* computed tomography, *SD* standard deviation

**Table 2:** Characteristics of the patient population who underwent cardiac CT for the evaluation of coronary artery disease; pre and post publication of the appropriate use criteria

	Pre-AUC n = 889	Post-AUC n = 1688	*P* value
Age (mean ± SD in years)	59.6 ± 12.1	59.7 ± 12.7	0.91
Female sex (%)	49.5	46.0	0.11
Cardiac risk factors			
Family history of CAD (%)	19.0	20.7	0.30
Active smoker (%)	15.3	13.5	0.21
Diabetes (%)	41.0	44.7	0.07
Dyslipidemia (%)	37.9	37.4	0.79
Hypertension (%)	19.4	18.5	0.62
Newly diagnosed systolic heart failure (%)	10.0	8.5	0.19
Prior history of cerebrovascular or peripheral vascular disease (%)	15.9	17.1	0.41
Chest pain			0.95
Typical (%)	20.9	20.4	
Atypical (%)	66.3	66.7	
No chest pain (%)	12.7	43.7	
Atrial fibrillation (%)	9.2	11.7	0.05
History of sustained ventricular tachycardia (%)	0.8	1.1	0.41
Abnormal prior stress test (%)	24.1	21.4	0.12

### Appropriate use of CCTA

**Fig. 2:**
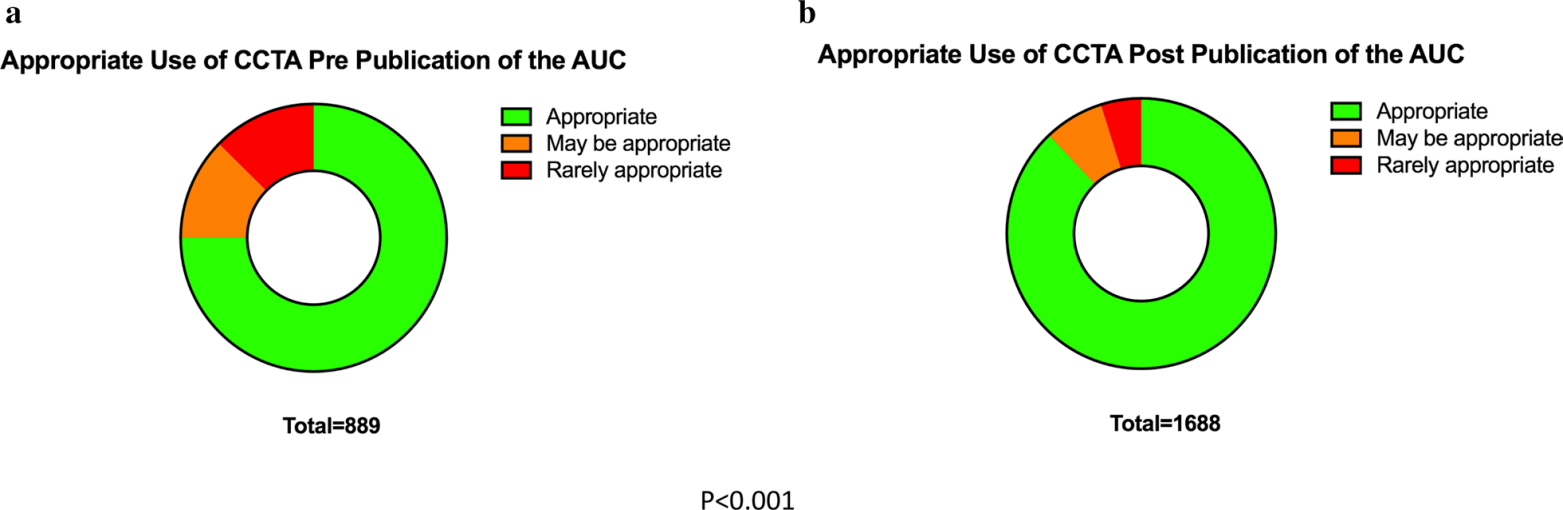
Classification of appropriate use (appropriate, may be appropriate, and rarely appropriate) of cardiac CTs performed for the evaluation of coronary artery disease before (**a**) and after (**b**) publication of the 2013 AUC

In the overall cohort, 83.5% of CCTAs were classified as “appropriate” based on the AUC, 9.0% of cases were deemed “may be appropriate” and 7.5% of cases were deemed “rarely appropriate”. In the pre-AUC publication group, only 75.0% of CCTAs were deemed “appropriate”, compared to 12.4% of CCTAs deemed as “may be appropriate” and 12.6% as “rarely appropriate”. In contrast, in the post-publication group (n = 1,88), 88.0% CCTs were deemed to be “appropriate”, while 7.2% were deemed to be “may be appropriate” and 4.9% were deemed to be “rarely appropriate” (*p* < 0.001, see Fig. 2).

### Symptomatic patients

There were 2,294 symptomatic patients in our cohort. These were patients who had either chest pain or an anginal equivalent. Of these, 1975 (86.1%) were deemed to be “appropriate”, 224 (9.8%) were deemed “may be appropriate” and 95 (4.1%) were deemed “rarely appropriate”. In the pre-AUC publication group, 530 patients (75.9%) were deemed “appropriate”, compared to 106 (15.2%) who were deemed “may be appropriate” and 62 (8.8%) who were deemed “rarely appropriate”. In contrast, in the post-AUC group, 1445 (90.5%) were deemed “appropriate”, 118 (7.4%) were deemed “may be appropriate” and 33 (2.1%) were deemed “rarely appropriate” (*p* value < 0.001).

### Asymptomatic patients

There were only 283 asymptomatic patients in our cohort. Of these, 177 (62.5%) were deemed “appropriate”, 7 (2.5%) were deemed “may be appropriate” and 99 (35.0%) were deemed “rarely appropriate”. In the pre-AUC group, 137 (71.7%) were deemed “appropriate”, 4 (2.1%) were deemed “may be appropriate” and 50 (26.8%) were deemed “rarely appropriate”. In contrast, in the post AUC group, 40 (43.5%) were deemed “appropriate”, 3 (3.3%) were deemed “may be appropriate” and 49 (53.3%) were deemed “rarely appropriate” (*p* < 0.001).

### Cost analysis

Prior to the AUC publication, 12.6% of CCTA scans were deemed rarely appropriate, translating to an annual cost of ~ $92.6 million upon extrapolation to institutions performing CCTAs in the United States. In contrast, post-AUC publication, only 4.9% of scans were classified as rarely appropriate, translating to an annual cost of ~ $36 Million. Therefore, when extrapolated to the Medicare population of the United States, we estimated a potential annual cost savings of approximately $57 million (see Table 3).

**Table 3:** Potential cost savings associated with the publication of the appropriate use criteria (AUC)

Absolute difference in rarely appropriate scans performed pre and post publication of AUC (%)	Unit cost per CCT (USD)	Estimate of number of CCTs performed annually in the Medicare population of the United States [19]	Potential annual cost savings (USD)
7.7	$432.36	1,700,000	~ 57 Million

*CCT* cardiac computed tomography

## Discussion

In our large retrospective study consisting of 2,577 consecutive patients undergoing CCTA, 83.5% of CCTAs performed were deemed appropriate. Following the publication of the 2013 AUC, the percentage of appropriate CT utilization increased significantly while the percentage of rarely appropriate utilization decreased significantly. There was no significant difference in patient characteristics when compared pre and post AUC publication. The decline in rarely appropriate scans translated to potential cost savings of approximately $57 million per year, when extrapolated to the Medicare population of the United States. Our results were driven by CCTAs performed on symptomatic patients, who accounted for approximately 89% of the total cohort. In the approximately 11% of patients who were asymptomatic in our cohort, the proportion of appropriate CCTAs declined after publication of the AUCs.

There have been a small number of previous studies examining the effect of AUC publication on appropriateness of CCTAs [9–13]. For example, a meta-analysis reported that while there was improvement in appropriate utilization of CCTA after publication of the AUC (from 37 to 55%), the overall rate of appropriate utilization remained relatively low [11]. The data included in the meta-analysis was generated from many parts of the world including the United States. One study included in the meta-analysis from the Mayo Clinic reported that only 27% of patients referred for cardiac CT were considered appropriate based on AUC criteria at that time [20]. In contrast, more contemporary data for cardiac magnetic resonance appropriateness by Kaushal et al*.* reported that 95.5% of cardiac MRIs were deemed appropriate. Similar to the Kaushal study, this paper also reported a significantly higher percentage of appropriate tests following AUC publication, in addition to a decline in rarely appropriate cases and an overall high number of appropriate CCTAs (83.5%) when compared to the older data. In this study, we found a higher proportion of appropriate CCTAs than what was reported in previous studies, although we found similar improvements in appropriateness in response to AUC publication. The relatively high number of appropriate CCTAs when compared to other studies may be in part related to the controlled access of the technology in our jurisdiction [21, 22]. Our findings may also be indicative of ongoing incremental improvement in physician ordering patterns, potentially as a result of iterative AUC publications. Although there are multiple factors that impact physician ordering patterns of non-invasive diagnostic testing, these results add to the growing body of evidence that supports the notion that publication of AUCs is associated with increased appropriateness of testing [23–25]. Furthermore, our study found potential cost savings of approximately $57 million per year arising from publication of the AUC. No study to our knowledge has previously determined cost savings associated with differing appropriate CCTA utilization in response to publication of the AUC. Our findings reporting potential cost savings associated with publication of the AUC are consistent with similar research performed on other imaging modalities, such as MRI [14, 25].

### Clinical importance

Ordering physicians in the United States will be required to consult the AUC when ordering advanced imaging tests such as CCTAs and cardiac MRI scans starting January 1st, 2021[5]. Our work highlights that after publication of AUC, appropriate utilization of CCTA is high in our large quaternary care centre. Interestingly, while our overall cohort and the symptomatic subgroup report higher appropriate utilization after publication of the AUC, appropriate utilization declined after AUC publication in our small asymptomatic subgroup. These results suggest that despite the impressive overall results, there remain areas for future improvement in the education of referring physicians and triage staff regarding the appropriateness of CCTAs in asymptomatic patients. Surveys aimed at evaluating physician consciousness and/or knowledge of the AUC before and after implementation of the mandatory consultation requirement, mentioned above, would be a valuable avenue for future research. Furthermore, our findings of $57 Million of annual potential costs savings that may be attributable to AUC publication indicate a potential real-world financial impact due to the publication of the AUC.

### Limitations

It is important to interpret the results of this study in the context of its limitations. First, in this study, CCTAs were scanned at one institution, potentially limiting results from being transferable to other jurisdictions. However, the institution is a quaternary care centre which receives patient referrals from four other community and academic hospitals and a wide array of outpatient clinics. Second, there are inherent limitations to this study due to its retrospective nature. For example, we did not evaluate appropriateness prospectively. The classification for each test was completed using retrospective patient data and occasionally by contacting the referring physician when needed. However, our classification methods were similar to those used in other studies with reported success rates for classifying patients > 95% [14, 15]. The retrospective nature of the study also translated to a lack of granularity in some clinical parameters, such as the processes used by referring physicians to access the AUC guidelines. Finally, traditional cost-effectiveness modelling was not appropriate for the design of this study. However, we used cost estimations using methods similar to those utilized in prior similar studies [19, 26]. Nonetheless, it is important to state that the cost savings reported in our study are estimates and not based on exact data (for example, the number of CCTAs performed annually in the United States is an estimated number because the exact number is not known).

## Conclusions

Our study consisting of approximately 2,500 consecutive CCTAs performed at a Canadian quaternary medical care institution describes an overall high rate of appropriate CCTA utilization coupled with an increase in appropriate utilization following the publication of the 2013 AUC. The AUC publication was also associated with significant decrease in the percentage of tests deemed rarely appropriate which translated to a potential estimated annual cost saving of approximately $57 million when extrapolated to the Medicare population of the United States.

## Data Availability

The datasets generated and/or analyzed during the current study are not publicly available due to the privacy policies at Sunnybrook Health Sciences Centre but may be available from the corresponding author on reasonable request.

## Abbreviations

AUC: Appropriate use criteria
CAD: Coronary artery disease
CCTA: Coronary computed tomography angiography
